# Speed encoding in the rat striatum

**DOI:** 10.1371/journal.pone.0334601

**Published:** 2025-10-27

**Authors:** Paulo H. Lopes, Lucas C. S. Tavares, Adriano B. L. Tort, Wilfredo Blanco

**Affiliations:** 1 Bioinformatics Multidisciplinary Environment (BioME), Federal University of Rio Grande do Norte, Natal, Rio Grande do Norte, Brazil; 2 Brain Institute, Federal University of Rio Grande do Norte, Natal, Rio Grande do Norte, Brazil; 3 Computer Science Department, State University of Rio Grande do Norte, Natal, Rio Grande do Norte, Brazil; Sorbonne Universite UFR de Biologie, FRANCE

## Abstract

The striatum plays a central role in motor control, yet how it dynamically represents variables such as locomotion speed, particularly across varying behavioral contexts, remains incompletely understood. Here, we investigated striatal encoding of locomotion speed in rats performing an automated T-maze task. We found that the activity of most (78%) analyzed striatal neurons— referred to as speed cells—was robustly correlated, either positively or negatively, with locomotion speed. This population included both putative medium spiny neurons (MSNs; 74%) and fast-spiking interneurons (FSIs; 82%). Speed-related activity was remarkably stable, showing no significant influence of elapsed time, cue type, spatial choice, or trial outcome. Additionally, positively correlated MSNs tended to precede speed changes, while positively correlated FSI activity typically followed, as did negatively correlated neurons for both types. This suggests distinct roles for different striatal cells in movement modulation. Speed cells exhibited strong modulation at movement onset and offset, yet also maintained correlations with speed throughout locomotion bouts. Finally, the firing rates of speed cells reliably predicted locomotion speed, outperforming non-speed cells and chance levels; decoding accuracy further improved when data from multiple neurons were combined, consistent with a population code. Together, these results demonstrate a robust, context-independent representation of locomotion speed in the rat striatum, driven by diverse cell types, and extends previous findings to a task with greater cognitive demands.

## Introduction

Motion is a fundamental aspect of animal behavior, underlying actions that range from complex navigational tasks to simple motor responses. The speed at which animals move is a critical factor in motor performance, influencing not only the efficiency of goal-directed actions but also playing a key role in survival-related behaviors such as hunting and predator evasion [1]. The striatum, the main input structure of the basal ganglia [2–4], receives sensory and motor signals and is essential for selecting and modulating actions [5]. Given its direct involvement in movement control [6], understanding how striatal neuronal activity relates to speed encoding is an important question. Previous studies have reported a linear correlation between locomotion speed and neuronal activity in the striatum of mice [3,7,8] and rats [9,10]. In the present work, we revisit these findings and further examine how the striatum represents speed and whether this encoding is influenced by external conditions.

The striatum contains a variety of interneurons, including cholinergic [11–13] and multiple GABAergic types [14–17]. Although relatively few, they shape the activity of the main projection neurons: the medium spiny neurons (MSNs), which constitute ~95% of all striatal neurons. In particular, fast-spiking interneurons (FSIs)—a distinct class of parvalbumin-expressing interneurons—play a critical role in feedforward inhibition by tightly regulating MSN activity [18]. Together, MSNs and FSIs form the core elements of striatal computation. While their anatomical and physiological properties are well characterized, their precise roles in encoding continuous behavioral variables, such as locomotion speed, remain less understood, particularly during cognitively demanding tasks.

To investigate the relationship between striatal activity and locomotion speed, we analyzed neuronal spiking in the striatum while rats performed an automated T-maze task with return arms. Our findings corroborate and extend previous research by demonstrating that (1) speed cells exist in the striatum; (2) these cells include both MSNs and FSIs; (3) the correlation between speed cell activity and locomotion speed can be either positive or negative; (4) other task-related factors—such as elapsed time, cue type, spatial choice, and trial outcome—do not affect the correlation between locomotion speed and striatal activity; (5) speed cell activity is modulated at the locomotion onset and termination; and (6) the firing rates of both MSNs and FSIs reliably predict locomotion speed.

## Materials and methods

We analyzed neuronal activity recorded from the rat striatum, originally collected by Oberto et al. [19] and made publicly available through the Collaborative Research in Computational Neuroscience data-sharing website (https://crcns.org/, Pfc-8 dataset) [20]. All procedures were approved by the Veterinary Services of Paris (n˚ B75-05-12). A complete description of the methodology, including behavioral protocols and surgical procedures, can be found in Oberto et al. [19]. For clarity and convenience, we provide below an overview of the experimental methods used in the original study. The data analyses were implemented using either Matlab (2020a) or Python (3.11) and the code is available in https://github.com/phb-lopes/Speed-encoding-in-the-rat-striatum-.git, https://github.com/phb-lopes/Speed-encoding-in-the-rat-.

### Tetrode implantation

**Fig 1:**
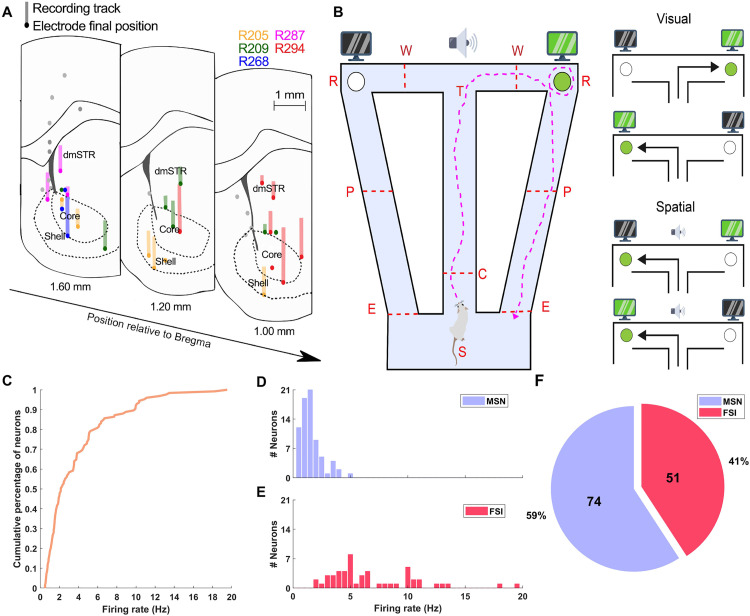
Experimental setup and neuron data overview. **(A)** Histologically verified tetrode locations mapped onto coronal atlas sections of the striatum, including the nucleus accumbens core and shell subregions. Each color represents a different animal (n = 5). Adapted from Oberto et al. [19]. **(B)** Schematic of the automated T-maze with return arms, illustrating an animal’s trajectory. Rats began each trial at the starting point S and received cues when crossing the photodetector **C.** They made left or right decisions at the choice point T, which were subsequently tracked by photodetector **W.** Correct choices triggered the release of reward (30 μL of 0.25% saccharin solution) at sites **R.** Photodetector P turned off the cue, and photodetector E indicated the end of the trial. Visual or spatial cues guided the animals’ decisions: in visual cue trials, the reward delivery site was indicated by the screens. In spatial cue trials, a tone indicated that the reward would be delivered in a fixed arm (selected as the animal’s non-preferred arm during pre-training), regardless of the visual cue. **(C)** Cumulative distribution of firing rates across all analyzed neurons. **(D)** Firing rate distribution of MSNs (medium spiny neurons; n = 74). **(E)** Firing rate distribution of FSIs (fast-spiking interneurons; n = 51). **(F)** Proportion of MSNs (blue) and FSIs (red).

Eight independently movable tetrodes (13-μm diameter tungsten wires, gold-plated to ~ 200 kΩ) were implanted in the ventral and dorsomedial striatum (AP 1.0–2.5 mm and ML 0.8–1.8 mm relative to bregma) of five Long-Evans rats (350–400 g). A miniature stainless-steel screw was implanted above the cerebellum to serve as reference and ground. Following surgery, the animals were allowed to recover for one week with *ad libitum* access to food and water. Afterward, tetrodes were advanced daily until they reached the target region (Fig 1A). At the end of experiments, animals received a lethal dose of pentobarbital and were intracardially perfused with saline followed by paraformaldehyde solution for subsequent histological verification of tetrode locations.

### Experimental design

Animals performed an automated T-maze task with return arms, in which the rules alternated between visual and spatial cues. At the beginning of each experimental session, an animal was placed in the central area of the maze (S label, Fig 1B). Once the animal self-initiated a trial, it could only move forward, crossing the central arm. Upon passing a photodetector located in the central arm (C label, Fig 1B), a cue was triggered to guide the animal’s choice at the decision point (T label, Fig 1B) toward the correct adjacent arm. A second photodetector (W label, Fig 1B) tracked the animal’s decision. In correct trials, a reward (30 μL of 0.25% saccharin solution) was delivered at the reward site (R label, Fig 1B). The cue remained active until the animal crossed a photodetector located in one of the return arms (P label, Fig 1B), and the trial ended when the final photodetector (E label, Fig 1B) was crossed. Trials proceeded without delay, irrespective of the previous trial’s outcome.

During visual cue trials, the cue was presented on a screen located in one of the adjacent arms, indicating the location of the reward. In spatial cue trials, the same screen was activated along with an auditory tone, but the reward was always delivered in the animal’s non-preferred arm, as determined during pre-training. In these trials, the animal had to ignore the screen and always head to the same rewarded arm. Transitioning between the two task rules (from visual to spatial or vice-versa) required the animals to achieve eight consecutive correct trials. This protocol was designed to test the animal’s ability to flexibly adapt to shifting task demands within a single session. Animals performed 15 sessions, totaling 1354 trials.

### Neuron type classification

Neuronal classifications were provided by the original authors, as described in Oberto et al. [19]. In summary, a k-means analysis distinguished putative medium spiny neurons (MSNs) from fast-spike interneurons (FSIs) based on spike waveform half-amplitude duration and trough-to-peak delay. Only neurons with an average firing rate greater than 0.5 Hz were included in our analyses, which corresponded to 125 out of 317 recorded neurons. Of note, while the observed proportion of putative FSIs (51/317 = 16% of all recorded units) is higher than expected based on anatomical estimates (~1–2%), such overrepresentation can occur due to their higher firing rates and greater detectability in extracellular recordings [21].

### Speed calculation

The dataset from Oberto et al. [19] included X-Y positions recorded throughout the sessions (sampling period = 0.0256 s). Instantaneous locomotion speed was estimated by first smoothing the X and Y time series using a 5-point moving average and then calculating the Euclidean distance between successive smoothed X-Y coordinates.

### Speed score and speed cell definition

Only epochs when the animals were performing the task were considered in the locomotion analysis. Periods when the locomotion speed was zero or unusually high (>60 cm/s) due to mistracking of position were also excluded. The remaining speed time series was binned into 1-second intervals with overlap of 900 ms (i.e., each 1-s bin represents the average speed over that time window). To compute the firing rate time series, the average firing rate of each neuron was calculated within the same 1-second bins. To obtain the speed score of a neuron, we first z-scored the locomotion speed and firing rate time series and then computed their cross-correlation. The speed score was defined as the peak value of the cross-correlation function, which, due to the z-scoring, corresponds to the Pearson correlation coefficient (Pearson’s r) at the corresponding time lag (referred to as the speed lag).

The speed score of each neuron was compared to a distribution of surrogate speed scores. A neuron was classified as a speed cell if its actual speed score exceeded ±2 standard deviations from its surrogate mean. Surrogate speed scores (n = 10000 per neuron) were calculated as the actual ones (i.e., from the peak of the cross-correlation between the z-scored time series), but with the firing rate time series randomly circularly shifted by 20% to 50% of the total analyzed time. To account for multiple comparisons across neurons, the resulting significance values were adjusted using the Benjamini–Hochberg false discovery rate (FDR) procedure.

Qualitatively similar results were obtained when employing smaller bin sizes of 250 and 500 ms, albeit with lower correlation coefficients (not shown).

### Analysis of speed cell stability across time, cue, spatial choice, and outcome

To evaluate the stability of the speed cell population over time, each session was divided into five trial blocks (with a minimum of 11 trials per block, given that the shortest session contained 59 trials). Speed scores were then computed separately for each trial block. To assess cue-related stability, speed scores were computed separately for trials involving either visual or spatial cues. Similarly, to evaluate speed coding stability based on spatial choice, speed scores were computed separately for trials in which animals chose either the left or right arm. Finally, trial outcome stability was analyzed by calculating speed scores separately for rewarded and non-rewarded trials.

To examine whether individual neurons were modulated by cue type, spatial choice, and trial outcome, we calculated the difference in the mean firing rate between trials belonging to each of the two states of each variable (i.e., visual vs spatial trials, right vs left choice trials, and rewarded vs non-rewarded trials). A surrogate distribution of firing rate differences between trial states was generated for each neuron by shuffling the trial labels (n = 10000 shuffles per neuron). A neuron was considered significantly modulated if its firing rate difference between trial states exceeded ±2 standard deviations from the mean difference of its corresponding surrogate distribution. This analysis was performed independently for each of the variables.

### Analysis of spiking activity at locomotion onset and offset

To identify locomotion onset, the speed time series was analyzed using 7-second sliding windows. Within each window, bouts of locomotion (i.e., locomotion onset events) were defined as follows: the animal’s speed was below 5 cm/s during the first 2 seconds, followed by an increase above 5 cm/s sustained for at least 50% of the subsequent 5 seconds. Similarly, locomotion offset events were defined when the speed was above 5 cm/s during the initial 2 seconds, followed by a decrease below 5 cm/s that persisted for at least 50% of the following 5 seconds. For each identified event, spiking activity was analyzed using a peristimulus time histogram (PSTH) with 0.1-second bins. PSTHs were normalized by dividing the firing rate by the mean firing rate across the event (such that the mean normalized activity equals 1).

### Linear decoding of speed

Linear decoding was implemented in Python. The input data consisted of firing rate and locomotion speed time series, both binned at 1-second intervals. To mitigate the impact of outliers, values from both time series were capped at their 95^^th^^ percentile (i.e., Winsorized). The time series were then smoothed using a Gaussian kernel (SD = 1 s). A scikit-learn pipeline incorporating polynomial features (degree = 3) and a linear regressor was used to predict locomotion speed from the firing rate time series. Cross-validation was performed by splitting the time series into 10 equal parts (9 epochs for training and 1 for prediction for each run), implemented using the KFold algorithm (n_splits = 10). Generalization performance (referred to as decoding accuracy) was then defined as the mean R2 score.

To establish a chance-level distribution of R2 scores, each speed cell’s firing rate time series was randomly circularly shifted by 20% to 50% of the total analyzed time. The same analysis pipeline described above was then applied to obtain a surrogate mean R2 score. This procedure was repeated 100 times per cell, and the average of these surrogate scores was considered the chance-level R2 score for that neuron. The overall surrogate distribution was obtained by pooling chance-level R2 scores across all speed cells.

Finally, to compute multi-cell decoding, only cells with an R2 score equal to or above 0.1 were included. We generated all possible combinations of 1–5 cells per session and used 10-fold cross-validation again to estimate generalization performance. In this analysis, each cell combination was considered an individual sample, irrespective of the session. As these cell combinations came from the same session, multicollinearity was expected; we therefore used Ridge regression (α = 1) to regularize the model. Surrogates were created using the same process as in single cell decoding, with the circular shifts applied simultaneously to all cells inside any given combination.

### Statistical analysis

Group data are presented as mean ± standard error of the mean (SEM). Statistical differences were assessed using unpaired, paired, and one-sample t-tests, as appropriate. For comparisons involving more than two conditions, repeated measures ANOVA were performed. When data did not meet the assumptions of parametric tests, non-parametric alternatives—including the Mann-Whitney U test and the Wilcoxon signed-rank test—were applied. Where multiple comparisons were conducted, Bonferroni or FDR correction was used to adjust the significance threshold. A p-value of < 0.05 was considered statistically significant.

## Results

To investigate the correlation between striatal neuronal activity and locomotion speed, we analyzed data from Long-Evans rats (n = 5) performing an automated T-maze with return arms (Fig 1). Fig 1B provides an overview of the task and cue system. During each trial, animals were required to cross the central arm and choose the adjacent arm according to the presented cue (either visual or spatial). The cue type alternated after the animal completed 8 consecutive correct trials.

A total of 125 neurons with firing rate >0.5 Hz were analyzed. As shown in Fig 1C, firing rates during task performance ranged from 0.5 to 19.5 Hz. Based on the classification by Oberto et al. [19], the analyzed sample comprised 74 putative medium spiny neurons (MSNs) and 51 fast-spiking interneurons (FSIs). MSNs typically fired at rates below 5 Hz, whereas FSIs exhibited higher and more variable firing rates (Fig 1D–1F).

### Striatal neurons correlate with locomotion speed

**Fig 2:**
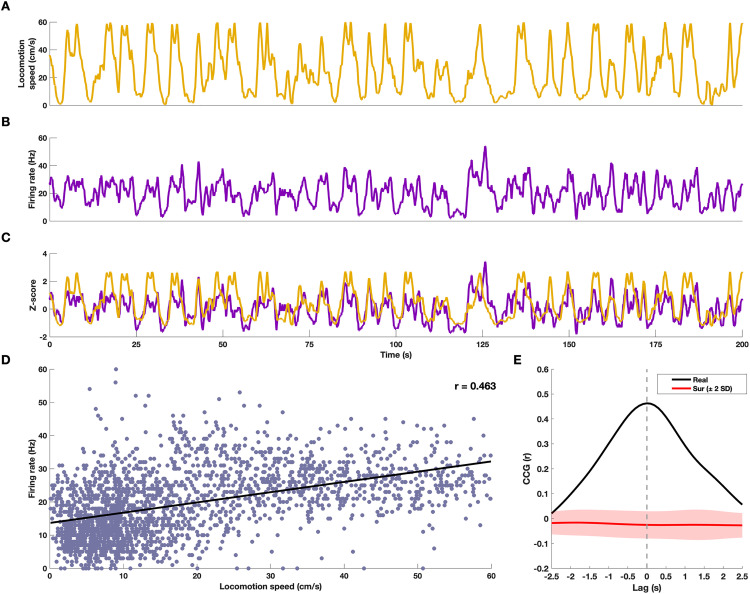
Striatal neuronal activity correlates with locomotion speed. **(A)** Time series of locomotion speed (cm/s) for a representative animal during performance of the T-maze task (1-second time bins with overlap of 900 ms). **(B)** Firing rate (Hz) time series of an example striatal neuron during the same session. **(C)** Same time series as in (A) and **(B)**, shown as z-scores for comparison. **(D)** Scatter plot of locomotion speed versus firing rate for the neuron in **(B)**, with linear regression line and Pearson correlation coefficient **(*r*)**. Only 15% of the data is shown. **(E)** Cross-correlogram (CCG) between z-scored firing rate and locomotion speed. The y-axis reflects the Pearson correlation coefficient (r) between the two time series at each lag. The black line represents the real data; the red line indicates the average of time-shifted surrogate data (see Methods), with the shaded area showing ± 2 SDs. The dashed vertical line marks zero lag. In subsequent figures, we refer to the peak CCG *r* as the *speed score*, and to the time lag at which this peak occurs as the *speed lag*.

We examined neuronal activity in the striatum during T-maze task performance. Fig 2A shows the locomotion speed time series of a representative animal over a 200-second interval. Locomotion speed varied dynamically between low and high values, with speed changes typically occurring around trial initiation and completion, turning points, and reward consumption.

Fig 2B presents the firing rate of a single striatal neuron recorded during the same 200-second interval. Note that the firing rate also fluctuated over time, closely tracking changes in locomotion speed. To highlight this synchronicity, Fig 2C displays both time series in z-score units, while Fig 2D shows their scatter plot along with the linear correlation (r = 0.463, p < 0.001).

Following previous work [22,23] striatal cells were classified as speed cells based on a speed score, defined as the peak of the cross-correlation between the z-scored time series of firing rate and animal locomotion speed (Fig 2E, black line). Notice that, due to the z-scoring, the speed score corresponds to the Pearson correlation coefficient at the time lag (hereafter referred to as ’speed lag’) where the peak occurs. To assess statistical significance, the actual speed score of each neuron was compared to a surrogate-based threshold derived from randomly time-shifted data after FDR correction (Fig 2E, red line and shaded area; see Methods). Under this framework, the example neuron in Fig 2 had a speed score of 0.463, which occurred at lag 0 and exceeded the statistical significance threshold.

**Fig 3:**
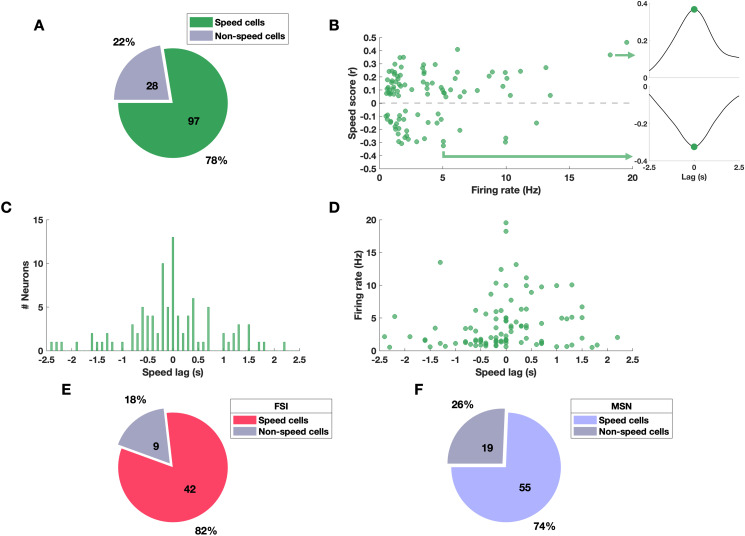
Characterization of striatal speed cells. **(A)** Proportion of striatal neurons classified as speed cells (green) and non-speed cells (gray). **(B)** Left: scatter plot of mean firing rate (Hz) versus speed score (*r*) for all speed cells (*n* = 97). Right: representative neuron with positive (speed score = 0.367, top) and negative correlation (speed score = −0.324, bottom). **(C)** Histogram of speed lag values **(s)**. **(D)** Scatter plot of speed lag versus mean firing rate. **(E)** Proportion of FSIs classified as speed cells (red) and non-speed cells (gray). **(F)** Proportion of MSNs classified as speed cells (blue) and non-speed cells (gray).

Fig 3 presents an overview of the speed scores and speed lags across the population of analyzed striatal neurons. Using the same criteria as described above, Fig 3A shows that 78% of neurons (n = 97/125) were classified as speed cells. These neurons exhibited a wide range of speed scores, from –0.32 to 0.46, revealing two contrasting subpopulations: one whose firing rates were negatively correlated with locomotion speed and another whose firing rates were positively correlated (Fig 3B, left panel). This contrast indicates that while some neurons increase their activity as the animal speeds up, others decrease their activity, potentially reflecting complementary roles in motor control. The right panel of Fig 3B illustrates representative neurons with positive (top) and negative (bottom) correlations. Notably, non-speed cells had firing rates below 11 Hz (mean ± SD = 3 ± 2.77 Hz, n = 28; data not shown), whereas speed cell firing rates reached up to 19.5 Hz (mean ± SD = 3.95 ± 3.87 Hz, n = 97). As shown in Fig 3C, most speed scores occurred at a near lag 0 (mean ± SD = −0.05 ± 0.88 s), suggesting that neuronal firing was closely coupled to locomotion speed. Positive speed lags indicate that changes in neuronal activity follow changes in speed, while negative lags indicate that firing rate changes precede them. Importantly, speed lag was not significantly correlated with firing rate (p = 0.13; Fig 3D). A similar proportion of FSIs (42/51, 82%) and MSNs (55/74, 74%) were classified as speed cells (Fig 3E and 3F).

**Fig 4:**
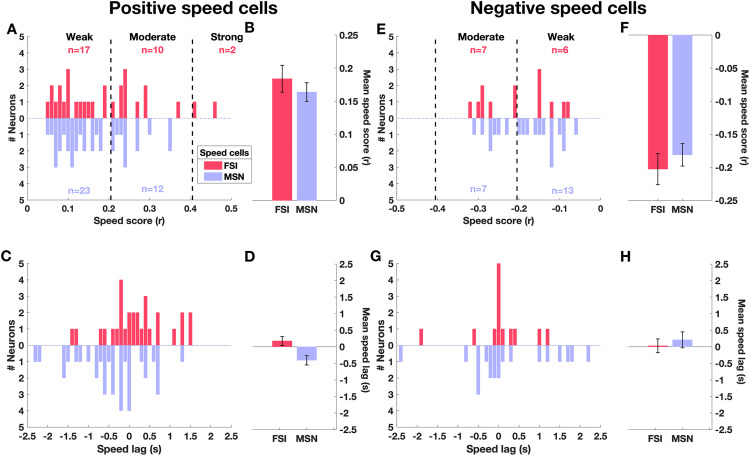
Characterization of positive and negative speed cells. **(A)** Histogram of positive speed scores separated by speed cell type (FSIs in red, MSNs in blue). Vertical dashed lines separate weak (<0.2), moderate (0.2 to 0.4), and strong (>0.4) speed correlations. **(B)** Mean speed score (± SEM) for FSI and MSN speed cells. **(C)** Histogram of speed lag values for FSI and MSN speed cells. **(D)** Mean speed lag (± SEM) for FSI and MSN speed cells. **(E-H)** Same as **(A-D)**, but for negative speed cells. In **(E)**, vertical dashed lines are set as weak (>-0.2), moderated (−0.2 to −0.4) and strong (<−0.4).

Fig 4A and 4E display histograms of positive and negative speed scores for FSIs (29/42, 69%; 13/42, 31%) and MSNs (35/55, 64%; 20/55, 36%), respectively. Across all speed cells, most neurons had weak (59/97, 60.8%) or moderate (36/97, 37.1%) correlation with speed. The mean speed score did not differ significantly between FSIs and MSNs for either positively (FSIs: 0.18 ± 0.02 [mean ± SD], MSNs: 0.16 ± 0.01, p = 0.4, unpaired t-test; Fig 4B) or negatively correlated neurons (FSIs: −0.2 ± 0.02, MSNs: −0.18 ± 0.01, p = 0.45, unpaired t-test; Fig 4F). Fig 4C and 4G depict the speed lag distribution for both cell types. Interestingly, in positive speed cells the firing rate changes in MSN tended to precede changes in locomotion speed (mean ± SD = −0.4 ± 0.14 s), while FSI firing rate changes followed them (0.17 ± 0.13 s; Fig 4D). In contrast, both FSI and MSN negative speed cells tended to follow changes in locomotion speed (FSIs: 0.03 ± 0.2 s, MSNs: 0.21 ± 0.24 s; Fig 4H). The speed lag difference between cell types reached statistical significance for positively correlated neurons (p < 0.005, unpaired t-test), but not for negatively correlated neurons (p = 0.6, unpaired t-test). Together, these results indicate that the majority of striatal neurons encode locomotion speed, with similar characteristics across FSIs and MSNs.

### Stability of speed cell activity across time, cue, spatial choice and trial outcome

**Fig 5:**
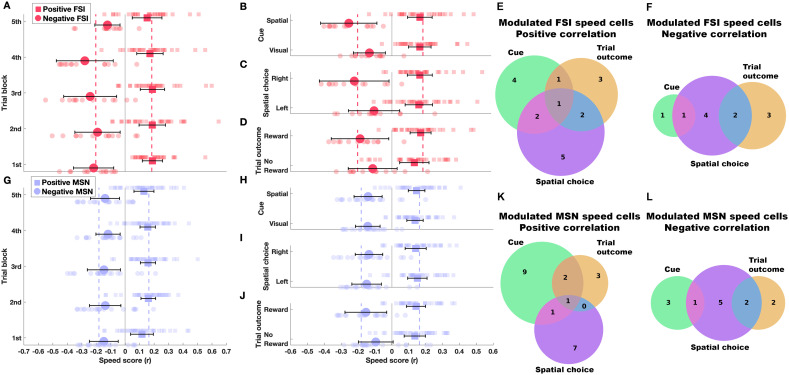
Striatal speed coding remains stable across time, cue type, spatial choice and trial outcome. **(A)** Speed score for FSI speed cells across trial blocks spanning the full task duration (n = 11 to 23 trials per block). Thin circles (positive correlation) and squares (negative correlation) show data for individual neurons; thick symbols with error bars represent the mean ± 95% confidence interval for each block. For reference, the vertical dashed lines indicate the average speed score computed over the entire task duration (as shown in Fig 4B and 4F). **(B–D)** Speed score for FSI speed cells grouped by cue type (visual vs. spatial; **B)**, spatial choice (left vs. right; **C)**, and trial outcome (no reward vs. reward; **D)**. **(G-J)** Same as **(A–D)**, but for MSN speed cells. In all cases, speed scores did not significantly differ from the session-wide score (one sample t-tests, *p* < 0.05). **(E-F)** Number of positive and negative FSI speed cells modulated by cue, trial outcome and spatial choice. Intersection areas show shared modulation. **(K-L)** Same as (E-F) but for MSN cells.

We next investigated whether the activity of striatal speed cells was influenced by external conditions. To this end, we computed and compared speed scores for trials subsets (see Methods). First, to assess whether the elapsed time within the task could affect speed-correlated firing, each session was divided into five blocks of trials, which were analyzed separately. As shown in Fig 5A and 5G, speed scores remained consistent across trial blocks for both cell types with no significant differences observed for positively (FSI: F(4,140) = 0.33, p = 0.85; MSN: F(4,170) = 0.88, p = 0.47, repeated measures ANOVA) or negatively correlated neurons (FSI: F(4,60) = 1.73, p = 0.15; MSN: F(4,95) = 0.14, p = 0.96, repeated measures ANOVA). These results demonstrate that the relationship between firing rate and locomotion speed is stable over time. Moreover, when comparing the speed score of each individual trial block against the overall score computed across the full session (as in Fig 4B and 4F), we found that no block-wise score was significantly different from the overall speed score (Fig 5A and 5G; one sample t-tests).

Similarly, further analyses of trial subsets revealed that neither the cue type (visual vs. spatial; Fig 5B and 5H), nor the spatial choice (left vs. right; Fig 5C and 5I), nor the trial outcome (non-rewarded vs. rewarded; Fig 5D and 5J) significantly influenced the speed scores (all p > 0.05, paired t-tests, Bonferroni corrected for three comparisons). Furthermore, the mean speed scores for each trial subset were not statistically different from the overall speed score computed for the full session (Fig 5B–5D and 5H–5J; one sample t-tests). In summary, these analyses demonstrate that the correlation between firing rate and locomotion speed remains stable regardless of external conditions.

Finally, to assess whether individual neurons are modulated by factors other than speed, we examined differences in their average firing rate between trial subsets defined by specific task features (see Methods). Fig 5E and 5F show the number of positively and negatively correlated FSI speed cells modulated by cue identity, trial outcome, or spatial choice, while Fig 5K and 5L present the corresponding data for MSNs. A subset of neurons was significantly modulated by a single factor (cue: 17/97, 14%; trial outcome: 11/97, 11.34%; spatial choice: 21/97, 21.64%). In contrast, fewer neurons were influenced by two factors (14/97; 14.4%), and less than 3% showed modulation by all three. These findings indicate that task-related variables also influence the firing of speed cells, although to a limited extent.

### Speed cell activity is modulated at locomotion onset and offset

**Fig 6:**
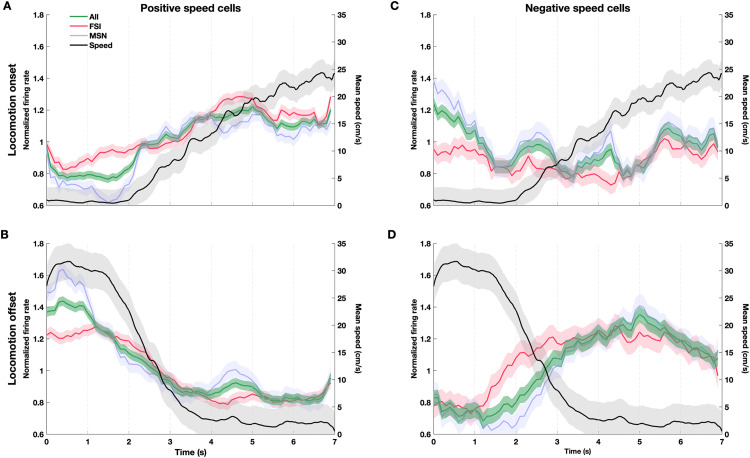
Speed cell activity is modulated at the onset and offset of locomotion. **(A)** Normalized firing rate of all positive speed cells (green, n = 64), and of FSI (red, n = 29) and MSN (blue, n = 35) speed cells also shown separately, aligned to locomotion onset (time = 0). The black trace shows average locomotion speed; shaded areas represent ±SEM. **(B)** Same as (A) but aligned to locomotion offset. **(C, D)** Same as **(A)** and **(B)**, respectively, but for negative speed cells (green, n = 33), and of FSI (red, n = 13) and MSN (blue, n = 20).

To investigate the spiking activity of striatal neurons around movement transitions, we analyzed firing rates aligned to locomotion onset and offset (see Methods). As shown in Fig 6A, the firing rate of positive speed cells increased sharply at the onset of locomotion, closely tracking the rise in locomotion speed–a modulation observed in both FSIs and MSNs. In contrast, negative speed cells showed a decrease in activity during the same locomotion onset events (Fig 6C).

A similar pattern was observed around locomotion offset: the firing rates of FSI and MSN positive speed cells decreased as animals slowed down and stopped (Fig 6B). On the other hand, negative speed cells increase their activity around locomotion offset (Fig 6D). These results indicate that speed cell activity is tightly coupled to movement transitions—positive speed cells ramp up their activity as animals begin to move and reduce it as they decelerate, whereas negative speed cells exhibit the opposite pattern, decreasing their activity with increased locomotion and increasing it as movement subsides.

### The firing rate of striatal cells predicts locomotion speed

**Fig 7:**
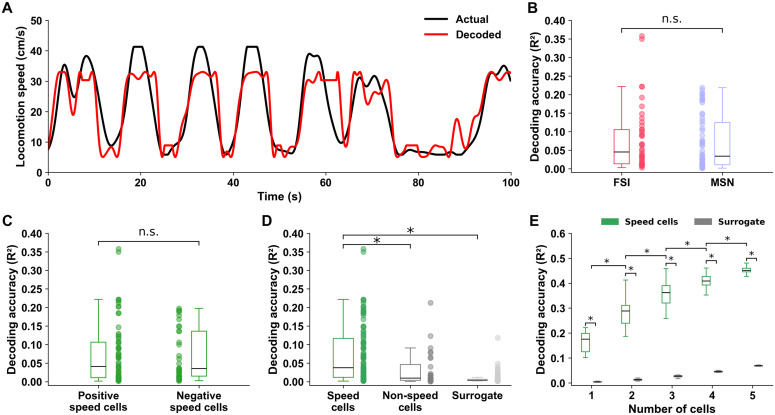
Linear decoding of locomotion speed from striatal activity. **(A)** Actual (black) and decoded (red) speed traces over a 100-second test window for a representative neuron with speed score = 0.462. Decoding was performed using linear regression. **(B)** Decoding accuracy (R2 score) for FSIs (red; n = 42) and MSNs (blue; n = 55) speed cells. No significant difference was found between neuron types. **(C)** Decoding accuracy (R2 score) for positive (n = 64) and negative (n = 33) speed cells. **(D)** Speed cells (green) show significantly higher decoding accuracy (*p < 0.001) compared to both non-speed cells (gray) and surrogate data (“chance levels”; light gray), in which the firing rate time series were randomly circularly shifted (see Methods). **(E)** Decoding performance improves with the number of speed cells and is consistently better than surrogate data. Boxplots and mean show decoding accuracy as a function of the number of neurons included in the model (*adjusted p < 0.001 for all pairwise comparisons).

To further validate our findings, we assessed how well striatal MSNs and FSIs encode locomotion speed by applying a linear decoding approach. Note that the R2 score used here is related to the decoding accuracy performance and not to the speed cell score (see Methods). Fig 7A shows the actual (black line) and decoded speed (red line) traces for a representative neuron, revealing closely matched fluctuations over time. At the population level, MSNs and FSIs exhibited similar levels of decoding accuracy (Fig 7B), with both groups showing high variance (R2 scores ranging from 0.001 to 0.358). There was no difference between positive and negative speed cell decoding accuracy (Fig 7C). In contrast, speed cells significantly outperformed non-speed cells in decoding locomotion speed (p < 0.001, r = 0.32, Mann-Whitney U-test; Fig 7D) and exhibited much greater decoding accuracy levels than expected by chance (p < 0.001, Wilcoxon signed-rank test; Fig 7D).

Finally, we investigated whether integrating activity from multiple neurons would enhance decoding accuracy. When restricting the analysis to neurons with an individual R2 score of at least 0.1 and evaluating all combinations of 1–5 cells per session, we found that decoding accuracy increased steadily with the number of neurons included (Fig 7E). All increments from 1 to 5 cells resulted in statistically significant improvements in decoding accuracy after Bonferroni correction for multiple comparisons. For instance, decoding with 2 cells was significantly better than with 1 cell (adjusted p < 0.001, r = 0.68, Mann-Whitney U-test), and this trend continued with increasing cell numbers (e.g., 5 vs 4 cells: adjusted p < 0.001, r = 0.61). The same trend holds when comparing against surrogate multi-cell combinations of the same size (adjusted p < 0.001, Mann-Whitney U-test).

## Discussion

We investigated the encoding of locomotion speed in the rat striatum during an automated T-maze task. Our investigation revealed that a majority (78%) of analyzed striatal neurons robustly encoded (positively or negatively) instantaneous locomotion speed. Following previous studies [22,23], we termed these neurons ‘speed cells’. Striatal speed cell activity was remarkably stable across varying task conditions, including elapsed time, cue type, spatial choice, and trial outcome, and reliably predicted locomotion speed. These findings extend previous research, largely conducted in mice during open field exploration [7] or treadmill locomotion [9], to rats performing a more complex cognitive task, and highlight a robust striatal representation of speed that appears largely independent of specific cognitive or contextual demands.

The role of the striatum in movement control is well established, with neuronal activity reflecting a range of motor actions, from subtle behaviors like head movements or licking to whole-body movements [3,4,7,24,25]. Both hierarchical feedback‐controller models and lesion or inactivation data indicate that striatal output must lead actual changes in movement speed by carrying a predictive “velocity reference” signal rather than merely reflecting sensory feedback. Turner & Desmurget [26] formalized this view, showing that movement-related changes in firing rate occur after motor cortex initiation but before or in parallel with globus pallidus internus output, supporting that basal ganglia outputs provide a feedforward gain signal that precedes kinematic execution (i.e., direction, amplitude and speed). Reinforcing this idea, Yin [27] proposed that cortico‐basal ganglia loops, particularly the sensorimotor striatum, implement a feedback controller for movement velocity, with dopamine modulating the gain of this control system.

Consistent with previous observations in mice [7,9,28], we identified speed-correlated activity in both major striatal cell types: putative medium spiny neurons (MSNs; 74% classified as speed cells) and fast-spiking interneurons (FSIs; 82% speed cells). Recent work has also shown that FSIs can represent the distance between the animal head and a target during a pursuit task, while MSNs encode self-velocity, with FSIs modulating MSN activity in a velocity-dependent manner [21]. This indicates that FSI activity may not only reflect locomotion variables such as speed but also play a causal role in shaping velocity encoding in MSNs through inhibitory control. Panigrahi et al. [29] suggested that striatal activity reflects ongoing or anticipated kinematic states but did not establish whether this activity directly drives movement. Bartholomew et al. [30] addressed this gap by using optogenetic stimulation of D1-expressing striatonigral neurons in mice and demonstrated a linear relationship between stimulation frequency and movement velocity. Their findings provide evidence that activation of the direct pathway is sufficient to modulate motor output in a graded, frequency-dependent manner, supporting the hypothesis that the striatum plays a causal and scalable role in setting movement speed.

While the electrophysiological classification does not resolve finer distinctions like D1- versus D2-expressing MSNs (i.e., direct and indirect pathway MSNs) or specific interneuron subtypes (e.g., parvalbumin-positive, cholinergic) identified in calcium imaging studies [7,28,31], we found that the overall strength of speed encoding, as measured by the speed score, did not significantly differ between the broader MSN and FSI populations. Moreover, both populations include neurons with positive (FSIs: 29/42, 69%; MSNs: 35/55, 64%) as well as negative correlation to locomotion speed (FSIs: 13/42, 31%; MSNs: 20/55, 36%). This distinct modulation is consistent with the diverse tuning profiles reported for MSNs in Panigrahi et al. [29], which included both significant positive and negative relationships between firing rate and movement speed, as well as with Sales-Carbonell et al. [24] that have shown the same for FSIs. Of note, given the compartmentalized organization of the striatum [32], future work should investigate whether speed coding is localized to specific striatal compartments. Additionally, potential differences in speed coding across distinct striatal subregions remain to be explored. In any event, our results suggest a widespread recruitment of diverse striatal cell types in representing ongoing locomotion speed.

Further analysis of the temporal relationship between neural activity and speed fluctuations—the ‘speed lag’—revealed that most speed cells (76.3%) exhibited peak correlations within ±1 second, indicating a tight coupling between striatal firing and ongoing movement. This is consistent with the time lags reported for functionally and anatomically segregated clusters of D1- and D2-MSNs in mice by Barbera et al. [28]. However, some neurons exhibited broader lags, ranging from −2.4 to +2.2 seconds, possibly reflecting a more complex behavior during the T-maze performance, which involves distinct phases of acceleration, deceleration, turning, and reward consumption. Interestingly, MSNs with positive correlations tend to precede speed changes (mean lag −0.4 s), whereas positively correlated FSIs tend to follow them (mean lag + 0.17 s). On the other hand, MSNs and FSIs with negative correlations generally follow changes in locomotion speed (mean lag 0.03 s and 0.21 s, respectively). Previous work has shown that D1- and D2-expressing MSNs have distinct functional roles [33,34]. In particular, activation of D1 MSNs promotes movement, whereas D2 MSNs suppress it [33]. Together, these patterns suggest differential functional roles: positively correlated MSNs may contribute to movement planning, while positively correlated FSIs and negatively correlated neurons of both types are more likely involved in real-time feedback.

Early study in the dorsolateral striatum of rats performing a treadmill task have demonstrated that these neurons can encode running speed often in conjunction with other variables like the animal’s position and time, suggesting integrative coding [9]. While our analysis focused specifically on locomotion speed rather than investigating multiplexing of other continuous intra-trial variables, we found no evidence that this encoding was affected by elapsed time, cue type, spatial choice, or trial outcome. This latter result aligns well with findings from Sales-Carbonell et al. [24] and Kim et al. [3], who, despite using different methodologies in mice, also reported that striatal neurons with speed correlations did not significantly differ in activity between rewarded and non-rewarded trials. In our sample, 16.3% of striatal speed cells were modulated by a single task-related factor (time, cue, or reward), 14.4% by two factors, and only 3% by all three. These findings suggest that task-related factors have a limited influence on the activity of speed cells, with locomotion speed likely serving as the primary driver of their modulation.

Striatal neurons have been shown to exhibit burst-like activity at the boundaries of learned actions (onset and offset), functioning analogously to ‘traffic light’ signals that gate movement initiation and termination [35,36]. Another line of investigation emphasizes a more continuous representation, wherein striatal neuronal firing rates are dynamically modulated throughout the execution of movement, reflecting ongoing kinematics [7,24,37,38]. The T-maze task, which demands speed changes during initiation, turning, reward approach, and trial completion, allowed us to examine both models. Consistent with the ‘traffic light’ concept, we observed clear modulations in the firing rates of speed cells at both locomotion onset and offset. Nevertheless, these same neurons also displayed a sustained correlation with speed throughout entire movement bouts. This suggests that what might appear as discrete start/stop signals could, at least in part, reflect the rapid changes in a continuously represented speed variable during acceleration and deceleration. Indeed, our results align with previous work by Sales-Carbonell et al. [24] and Fobbs et al. [7], who also found that neurons exhibiting continuous speed correlation could show prominent activity changes around movement transitions without necessarily forming distinct ‘start’ or ‘stop’ cell populations. Consistent with this view, Panigrahi et al. [29] showed that both direct D1- and indirect D2-MSNs in the dorsomedial striatum exhibit sub-second activity changes that anticipate accelerations and decelerations of running speed. They concluded that striatal MSNs carry an online “vigor” signal encoding instantaneous speed, with dopamine essential for setting the gain of this representation in real time.

Further supporting the role of the striatum in continuously encoding speed, our linear decoding models accurately predicted instantaneous locomotion speed from the firing rates of individual speed cells—regardless of cell type—significantly outperforming non-speed cells and chance levels. These results are consistent with findings from Fobbs et al. [7] and Barbera et al. [28], who also reported reliable speed decoding, although using different methodologies. Notably, decoding accuracy progressively improved when combining information from multiple speed cells. This suggests that while individual neurons carry significant speed information, the striatum likely employs a population code to represent locomotion speed with higher fidelity. Such distributed encoding is a common feature in motor-related brain areas and may contribute to the flexibility and robustness of motor control [39–41].

In conclusion, our findings demonstrate that the striatum contains a robust representation of locomotion speed encoded by both MSNs and FSIs. This representation persists across varying cognitive and contextual conditions and supports accurate reconstruction of movement speed from neural activity. Understanding how the striatum encodes kinematic variables like speed is essential for a complete model of basal ganglia function and may inform the study of movement disorders that impair motor regulation.
